# The Role of Social Support on Self‐Management of Gestational Diabetes Mellitus: A Qualitative Analysis

**DOI:** 10.1111/jmwh.13782

**Published:** 2025-06-27

**Authors:** Tazim Merchant, Julia D. DiTosto, Maria Gomez‐Roas, Brittney R. Williams, Charlotte M. Niznik, Joe Feinglass, William A. Grobman, Lynn M. Yee

**Affiliations:** ^1^ Division of Maternal‐Fetal Medicine, Department of Obstetrics and Gynecology Northwestern University Feinberg School of Medicine Chicago Illinois; ^2^ Department of Obstetrics & Gynecology Mayo Clinic, Rochester Minnesota; ^3^ Department of Biostatistics, Epidemiology, and Informatics Perelman School of Medicine, University of Pennsylvania Philadelphia Pennsylvania; ^4^ Division of General Internal Medicine Northwestern University Feinberg School of Medicine Chicago Illinois; ^5^ Division of Maternal‐Fetal Medicine, Department of Obstetrics and Gynecology The Warren Alpert Medical School of Brown University Providence Rhode Island

**Keywords:** gestational diabetes, lifestyle, pregnancy, self‐management, social support

## Abstract

**Introduction:**

Given the rising incidence and importance of treatment adherence for gestational diabetes (GDM), this study aimed to examine patient perspectives on social support's role in gestational diabetes (GDM) management during pregnancy and early postpartum.

**Methods:**

This is an analysis of qualitative data collected during a feasibility randomized controlled trial of postpartum patient navigation for individuals with GDM. Participants completed semistructured interviews at 4 to 12 weeks postpartum on their experiences with GDM as well as facilitators and barriers to its management, including social support. Data were analyzed using the constant comparative method.

**Results:**

Of 38 participants, 55% reported a family member or friend with a history of GDM, type 1, or type 2 diabetes mellitus. The analysis identified 4 themes: (1) communal support, (2) indirect GDM support (ie, removing logistical barriers to care), (3) direct GDM support, and (4) barriers of social support (eg, stigma), which impeded disease understanding. Participants identified communal support as a catalyst for lifestyle change and a source for advice and emotional support. Indirect support included transportation, childcare, and work accommodations. Direct support included providing healthy food, giving insulin shots, or providing accountability.

**Discussion:**

Harnessing social support may be a key strategy to activate and sustain lifestyle and self‐management skills in GDM and should be considered in intervention development.

## INTRODUCTION

Gestational diabetes mellitus (GDM) is an increasingly common pregnancy complication, with its incidence rising from 47.6 to 63.5 per 1000 live births from 2011 to 2019 in the United States.^1^ Risk factors for GDM include advanced maternal age and obesity, both of which are growing in prevalence.^1^, ^2^, ^3^, ^4^ GDM is associated with significant short‐ and long‐term health risks for both the pregnant individual and their offspring. People with GDM are at elevated risk for experiencing other pregnancy complications such as hypertensive disorders of pregnancy or cesarean birth, and are at higher risk of developing type 2 diabetes mellitus (T2DM).^4^, ^5^ Neonatal complications are also more frequent among people with GDM, including increased incidence of macrosomia, preterm birth, and long‐term metabolic sequelae.^1^, ^4^ Thus, effective management of GDM through medical nutrition therapy, activity, and, when indicated, pharmacologic intervention, is essential to optimizing maternal and child health outcomes.^4^ However, adherence to treatment recommendations can pose challenges to pregnant individuals due to factors such as limited health literacy, difficulty implementing lifestyle changes, psychosocial stress, and competing burdens.^6^, ^7^, ^8^ Thus, it is necessary for clinicians to understand obstacles to and facilitators of GDM self‐management in order to support patients and optimize their health.

One facilitator of self‐management may be social support. Social support, defined as the assistance and protection given to others through tangible (eg, checking glucose) or intangible (eg, emotional support) means, usually by family or friends, can play a key role in diabetes self‐management.^9^ The role of social support in T2DM has been well documented.^9^, ^10^, ^11^, ^12^, ^13^ Social support can help control blood glucose levels and hemoglobin A1C and promote adherence to lifestyle changes and treatments.^12^, ^14^, ^15^ In contrast, other studies have shown that certain actions taken by social support networks can negatively influence glycemic control, particularly for adults with limited health literacy.^16^, ^17^

**QUICK POINTS**

✦ Social support is a key social determinant of health that has been linked to a difference in health outcomes.
✦ Adherence to treatment recommendations in gestational diabetes can be difficult in the setting of limited health literacy, psychosocial stress, and competing burdens
✦ Patients with gestational diabetes shared that communal, indirect, and direct support can aid lifestyle change and self‐management
✦ GDM‐focused patient education materials and interventions may be improved by leveraging social support's benefits while preventing obstructive behaviors or misconceptions

The role of social support for people with GDM is less understood. Some data suggest social support may be helpful in overcoming barriers to health‐promoting behaviors in GDM and that less social support is correlated with less self‐efficacy and higher body mass index.^18^, ^19^, ^20^, ^21^ Individuals have also been shown to be receptive to postpartum peer social support programming.^22^ However, perspectives among people with GDM regarding when social support is helpful or harmful have not been well studied.

Social support theory describes 4 dimensions of social support: (1) appraisal support (eg, validating the appropriateness of food choices), (2) informational support (eg, assisting with problem solving, such as how to best control glucose levels), (3) instrumental support (eg, providing tangible items or services such as directly preparing food), and (4) emotional support.^9^, ^10^ Better characterization of the types of social support individuals perceive as helpful can provide insight into how to best construct interventions to effectively mobilize social support networks and harness its benefits. This understanding is especially important among individuals with GDM because increased stigma and psychosocial concerns underscore the need for a holistic approach to support treatment adherence and lifestyle change.^8^, ^23^, ^24^ Thus, we aimed to better understand perceptions of social support's role in GDM self‐management during pregnancy and the early postpartum period.

## METHODS

### Research Design

This qualitative investigation occurred as a secondary analysis of data from the Sustaining Women's Engagement and Enabling Transitions after GDM (SWEET) study, a feasibility randomized controlled trial (RCT) of postpartum patient navigation for individuals with GDM.^25^ All participants were asked to complete an interview upon exit from the trial. Interviews were designed to both solicit feedback about the intervention under investigation as well as to explore participants’ GDM‐related experiences. A qualitative approach was chosen to glean specific narrative insights from participants on the role of social support and how participants integrate its benefits and are affected by potential barriers.

### Feasibility RCT

In the parent SWEET study from which qualitative data for this study was derived, individuals were recruited from a large, urban academic medical center in Chicago between December 2020 and July 2021. Pregnant or postpartum individuals with GDM who were giving birth at or after 30 weeks' gestation were eligible for participation. Additional eligibility criteria included being age 16 or older, speaking English or Spanish, and receiving care at the study site. In the RCT, participants were recruited after 35‐weeks’ gestation or, if birth occurred between 30‐ and 35‐weeks’ gestation, at the time of postpartum hospitalization. All participants provided written informed consent upon enrollment. They were then randomly assigned to either the intervention group with a postpartum patient navigator or to usual care. The aim of the SWEET study was to investigate the feasibility of a patient navigation intervention designed to improved diabetes‐related postpartum health outcomes. The study detailed in this article reflects a secondary analysis of interview data collected in the parent study to examine the role of social support in GDM management. The parent SWEET study was approved by the Northwestern University institutional review board and registered on ClinicalTrials.gov (NCT04583839).^25^

### Data Collection Procedures

Research team members were trained to perform in‐depth interviews in English or Spanish at the postpartum visit (4‐12 weeks after giving birth) for all participants in the SWEET RCT. Research team members were non‐health care professionals who were not involved in the clinical care of participants. A semistructured interview guide was designed to facilitate an open‐ended, nonjudgmental conversation with participants regarding their experiences with diagnosis and management of GDM, including facilitators and barriers to managing GDM (Table 1). Interviewers explicitly inquired about participants’ social support via direct inquiry (eg, family members’ responses to the participant's GDM diagnosis, support systems to achieve goals), as well as through probes about topics raised by participants. Interviews were 30 to 45 minutes in length. Participants were compensated after completion of the interview with gift cards.

**Table 1:** Semistructured Interview Content

Content Categories	Description
Experience with GDM diagnosis	Understanding of and familiarity with GDM Whether friend or family had GDM and how their experiences were similar or differed from participants How participant felt about their diagnosis, and sharing it with friends and family
GDM care	Barriers to managing GDM Self‐management practices and resources People who helped with care navigation
Prevention of type 2 diabetes mellitus	Habits participants want to continue integrating Postpartum glucose tolerance testing Experience with weight gain during pregnancy and weight loss goals Breastfeeding and preventing diabetes Weight goals and participants’ support systems to help achieve them

Abbreviation: GDM, gestational diabetes mellitus.

### Data Analysis

For the purpose of this thematic analysis, the study team focused on qualitative coding of transcripts on social support‐related themes using the constant comparative method.^26^, ^27^ Interviews were audio‐recorded and transcribed verbatim by a professional service. Transcripts were then uploaded into Dedoose, a web‐based tool for qualitative analysis, for analysis via the constant comparative method.^26^, ^27^, ^28^ The study team developed a codebook based on the interview guide and preliminary review of transcripts. Additional subthemes were added with continued transcript review to ensure saturation. Additional themes were applied using the framework of social support theory, which describes how family, friends and others can provide support addressing emotional, education, and other needs.^9^, ^10^ The investigative team collectively provided feedback on codes during this process to ensure adequate organization, negation, and agreement of themes. Two research assistants with training in qualitative coding applied the final set of codes systematically and independently to the full data set using Dedoose to code transcripts electronically. The investigative team reviewed code application after transcript analysis to ensure consistency. Through this collaborative, discussion‐based analytic process, social support‐related themes and subthemes emerged and were then more specifically analyzed via the lens of social support theory.^9^, ^10^

### Reflexivity Statement

Authors included in this article contributed in several ways including study design, data analysis, interpretation, and synthesis. The senior author (LMY) is a cisgender female person of color maternal‐fetal‐medicine physician who provides health care to a diverse patient population and has extensive training and research background in public health. She was responsible for conceptualization of this study and overseeing its design. The lead author (TM) is a female cisgender queer person of color who directly led and was responsible for data analysis, interpretation and synthesis. Data collection was performed primarily by the second author with assistance with recruitment by the fourth author, and both authors underwent training to conduct unbiased, person‐centered interviews. Secondary analysis was performed by members of the research team who are both persons of color (first and third authors), with findings reviewed and commented upon by the larger team inclusive of all authors. All members of the team are female identifying apart from one author. Although efforts were made to avoid bias in interviews and data analysis and interpretation, our roles and identities may have influenced our perspective on these aspects of the study. The consolidated criteria for reporting qualitative research were consulted and reviewed in the preparation of this article.^29^

## RESULTS

Of 40 participants in the SWEET study, 38 completed qualitative interviews and were eligible for this analysis. A majority identified as persons of color (31.6% non‐Hispanic Black and 44.7% Hispanic). Half had public insurance for antenatal care, and more than half (55%) reported a family member or friend with a history of either GDM, type 1, or type 2 diabetes (Table 2).

**Table 2:** Participant Demographic Characteristics (n = 38)

Characteristic	Value^a^
**Age, mean (SD), y**	33 (6.5)
**Race and ethnicity, n (%)**	
Non‐Hispanic White	12 (31.6)
Non‐Hispanic Black	12 (31.6)
Hispanic/Latinx	17 (44.7)
Asian	5 (13.2)
American Indian/Alaska Native	2 (5.3)
Other	12 (31.6)
**Language, n (%)**	
English	36 (94.7)
Spanish	2 (5.3)
**Insurance, n (%)**	
Commercial	18 (47.4)
Medicaid	19 (50.0)
Military	1 (2.6)
**Approximate yearly income, before taxes, n (%)**	
Under $10,000	5 (13.2)
$10,000‐25,000	5 (13.2)
$25,001‐50,000	11 (28.9)
$50,000‐100,000	3 (7.9)
$100,000‐200,000	5 (13.2)
Over $200,000	7 (18.4)
Don't know or decline	2 (5.3)
**Time of recruitment to the parent trial, n (%)**	
Antepartum	35 (32.1)
Postpartum	3 (7.9)

Analysis revealed 4 major themes regarding social support's role in GDM: (1) the role of communal support, (2) indirect GDM support, (3) direct GDM support, and (4) barriers of social support. Each theme and subtheme, and their connection to social support theory domains, are discussed below and in Table 3.

**Table 3:** Themes and Subthemes Regarding the Role of Social Support in GDM Management

Theme and Subtheme	Representative Quotes
**Communal Support**	
Communal emotional support	It was important for me to know, oh my god—I'm not the only one going through this. Yes, the dietician, the doctor, and the nurses are telling you to eat blah blah blah blah blah at a certain time. A lot of women are going through the same thing. So, just reading their comments was helpful to me.
Support groups	I found it somewhat helpful, just seeing that it wasn't like just me and seeing what other experiences other people were going through. And then specifically, like, if I had small questions that wasn't like medical concerns, I could ask that group. Like what kind of food are they eating, or you know if they had this reading, like specifics like that, like what it meant and stuff like that, but yeah, it was helpful in that sense.
Communal advice	I'd always go to [my dad] and be like “should I eat this, is it okay to eat this? Like what raises your blood sugar, what lowers it?” I definitely asked him for a lot of advice.
Communal lifestyle changes	How do I plan? Just I guess, motivating everybody. Because I feel like if everybody is on the same page as me, it'll be easier for me. Like okay if we can eat this today, like this healthy dish you know, you'll like it and you know they want to eat it, I feel like happier. Like okay I don't have to force you, you know? You want to make that choice with me, you know to be healthy with me. Just kind of motivate my family and like my house, and it will motivate me as well to continue eating healthier.
**Indirect Support**	
Transportation	Driving me here, waiting for me. Those [medical] appointments, [my husband] waited for me, parked outside, [would] get up early or come late if I had to do something here.
Childcare	Yeah. I mean I had the childcare issue, which is exactly why I had to reschedule. Just because with COVID, I'm not going to have just anybody watch my children, especially since I have a brand new baby, so I had to have my mom come from out of town to watch them, so that was a challenge for me.
Work accommodations	They accommodated as much as they could. And then also with my experience of having GDM, I used the bathroom so often, so, so often. Took a lot of bathroom breaks, having to track numbers, making sure I'm getting all the snacks and meals in. That part made it hard throughout the workday, but they did try to accommodate as much as they could.
**Direct Support**	
Giving shots or checking glucose	Yeah. I prefer someone to do it. How my mother‐in‐law does hers is she wants someone to put the medicine in her arm. She can't do it herself. She hates needles.
Providing healthy food	My parents…they're making me learn how to cook healthier dishes so all of that has been helpful…everything's been healthy that my mom cooks.
Accountability for medical care or goals	Me and one of my closest friends were in a Fitbit challenge. And we do challenges every week to see where we at, and then we motivate each other—like if we notice that we see each other's Fitbit results—so if we're lower than what the expected goal is, she'll text me and say, “why are you not walking,” or “what's going on,” or “did you sleep good.” We do a wellness checkup on each other.
Motivator: others’ success with their own goals	My mom… just seeing her and her transformation within just a couple months. She started last year, but she's really been going hard at it since like February, and she looks really good.
Motivator: impact of illness on fetus and living children	I thought it was going to affect the girl too; that she was going to have diabetes too. That's why I tried to take care of myself.
**Barriers of Social Support**	
Limited knowledge of GDM	But I think the difficult thing wasn't for me, it's just for everybody else around me. There's certain things I couldn't eat, and they would still offer them to me. Or you know, like, my husband would be like, “oh, I got something to eat,” and then he would bring something that I couldn't eat. I'm like, “I can't eat that. I can't eat that. You know already what I can't—“ and he would just forget.
Stigma	I didn't really love discussing it. I feel like there was a little bit of, initially like kind of shame around it…I felt very much like this was something I'd done wrong.
Excessive concern	But it was a lot. Like a lot lot. Everybody was making it way more urgent too, like “did you take your sugar,” “what's your sugar?” Everybody's texting me or reminding me, or “stop stressing yourself so much, what was your blood pressure…”

Abbreviation: GDM, gestational diabetes mellitus.

### Role of Communal Support

Participants cited communal support (ie, helping individuals feel less isolated), whether it was from family members with diabetes, or others with GDM, as a positive presence. Four subthemes included: (1) communal emotional support, (2) support groups, (3) communal advice, and (4) communal lifestyle changes.

Several participants reported feeling communal emotional support when they interacted with different types of communities (eg, family, friends, online forums). One participant shared this sentiment about a helpful interaction with her mother:

So, I lost a lot more weight, and this time I moved in with my mother and I could only eat her food…I think because of my mother, I was feeling a little bit better with this pregnancy, if that makes sense, than the first one. Because I had support and because I physically had people there helping me.

Support groups were particularly helpful to participants who did not have family members with diabetes. The sense of community gained from support groups was a recurring theme. This was affirmed by one participant:

I didn't have any friends or family members who'd had it, so I joined like a Facebook group and met some people through that who had gestational diabetes. Talking to them and hearing about their health experiences…[people] who were still struggling with the same thing…I think that was helpful.

Participants also reported receiving practical communal advice, a form of informational social support,^9^, ^10^ on how to adhere to lifestyle changes. Advice ranged from how to check blood glucose levels (“how to use the needle”) to the types of foods to eat, and tips to keep blood sugar within range.

For some participants, communal support (ie, from multiple individuals and/or groups) provided accountability for communal lifestyle changes, an extension of appraisal support.^9^, ^10^

### Indirect GDM Support

Indirect support (ie, removing logistical or other barriers so participants can focus on care) primarily included transportation, childcare, and work accommodations. A few participants noted that family members or a partner would help with transportation, driving them to and from appointments. One participant shared: “My boyfriend was pretty flexible about like having to bring me here because it was important to him that I get checked and that the baby was okay.”

Providing childcare was also a way in which social support helped participants adhere to their appointments. This support is significant given that GDM requires more time‐intensive health care with often weekly medical encounters. One participant affirmed:

“Childcare maybe because I had to have someone watch while I had, I had to come to the doctor extra. You know, with pregnancy I have to come like once a week to be tested and taken care of. When…other people didn't have to in their pregnancy.”

Participants commented that childcare support offered them time to pursue healthy lifestyle activities. One participant shared, “If [my partner] can watch the baby, I can go like run maybe… I think that's important.” A third type of indirect support, work accommodations, is described in Table 3.

### Direct GDM Support

Participants also received instrumental or appraisal social support that directly assisted or motivated them to make GDM‐specific lifestyle changes and adhere to medical treatment.^9^, ^10^ Direct support subthemes included help checking blood glucose, providing healthy food options, promoting accountability, and providing motivation.

First, a small number of participants expressed concerns about checking their blood glucose, citing fear of needles or the timing of glucose measurements as barriers that were sometimes ameliorated by social support. One participant shared, “I hate needles, so I was afraid. So, my husband did it for me. I would only have to use it [insulin injection] before bed, so he would do it for me.”

Others shared that a partner or parent would often provide healthy food, either by cooking healthy dishes, or grocery shopping for items that aligned with an appropriate GDM diet. One participant indicated this type of support helped her form long‐term habits:

My diabetes was very well controlled because my mother was cooking so when I told her I had gestational, she told me, ‘Oh, no.’ She cooked for me separately, like vegetables, salads, chicken, fish. She helped me a lot to take care of myself…in fact, as my father has diabetes too, she took care of both of us. Actually, I got used to eating vegetables and salads. I drink a soda, but sometimes once a week. You get used to eating healthy.

Similarly, many participants discussed the role social support played in promoting accountability for medical care and goals. For instance, friends who did not exercise chose to do so in order to encourage participants. Some participants cited their children as a driving force to stay active because playing outside led to increased physical exercise.

The motivation subtheme was described in 2 manners. First, many participants were motivated by encouragement from friends and family members who had successfully achieved their own lifestyle goals. For example, some participants reported that loved ones directly shared their weight loss or exercise experience, which helped sustain the participants’ own health behaviors. Other participants noted being motivated to maintain their healthy lifestyle changes after observing friends or family members achieve their own weight loss or exercise goals. Second, participants were encouraged by discussions with health care providers or family members about the potential effects of GDM on the fetus and children. One participant noted that conversations with her physician and family prompted her to entirely shift her health behaviors, illustrating how direct support played a role in her motivation:

I wasn't following my recommendations that I was supposed to do, so then my first thought was what if something is wrong with my baby because I didn't do what I was supposed to do. So that's what made me eat better.

### Barriers of Social Support

Despite the positive attributes of social support when managing a pregnancy with GDM, participants reported certain behaviors of friends and family members can detract from well‐being and care. Analysis demonstrated 3 barriers of social support: (1) limited knowledge of GDM, (2) stigma, and (3) excessive concern.

Limited knowledge of GDM sometimes made it difficult for participants to share their experience with family and friends. Individuals struggled with having to explain their disease, its management, and the cause. One participant noted how limited knowledge of GDM contributed to awkward social interactions:

“You would go to…a dinner…and you would say, ‘well, I just had my insulin shot, so I have to eat. We can't [wait] for the waiter, or all of you to have your orders ready’…So it makes you feel like a little weird…”

Similarly, stigma was emphasized as a barrier by several participants, who felt shame surrounding their diagnosis and shared how family or friends reinforced the feeling that GDM was the participant's fault. As a result, many chose to not disclose their diagnosis. One participant stated:

Because of COVID, though, I didn't really have a lot of interaction with a lot of other family members, other than my mom, but I could imagine…some people thinking it's something I had control over, or actually I guess my mom had some of that. She thought it was something I had control over. And I had to explain to her that it wasn't under my control.

A few participants also relayed experiences in which well‐intentioned accountability provided by social support went too far. Family members demonstrated excessive concern, sending reminders or imposing strict boundaries to the point that the participant felt increasingly stressed or controlled by the people intending to provide support. One stated:

He [my partner] was basically micromanaging what I eat, and once he sees me with a cookie in my hand, he'll grab it and say ‘no, don't hurt the baby, don't hurt my baby,’ and I'm like ‘you're overdramatic…’ It was annoying, to be honest with you.

## DISCUSSION

This study found that social support was perceived to be helpful in participants’ management of GDM via 3 key avenues: communal, indirect, and direct support. Individuals often asked advice on diabetes self‐management from friends and family with diabetes, support groups, or online communities. Participants received direct and indirect support with GDM self‐management tasks that were unique to pregnancy (eg, support for attending antenatal testing) and those more general to diabetes. However, this study also underscored that members of one's social support network may have little GDM knowledge or contribute to stigma of or overwhelm participants in relation to GDM.

As described above, the 4 dimensions of social support include appraisal, informational, instrumental, and emotional support.^9^, ^10^ Our findings align with extant work on social support theory in other health domains. For instance, the theme of communal support encompassed the dimensions of emotional support by creating a sense of community around patient experiences (eg, via support groups). Direct support included aspects of appraisal support by providing accountability around medical care and goals, and instrumental support (eg, glucose‐checking). Indirect support included forms of instrumental support.^9^, ^10^ Our findings are also consistent with documented perspectives on the role of social support in coping with stress and in promoting self‐efficacy and self‐regulation.^7^, ^30^, ^31^, ^32^

Our findings regarding social support limitations (eg, stigma), have also been noted in the nonpregnancy‐related literature.^16^, ^17^ As Mayberry and Osborn demonstrated among individuals with T2DM, behaviors we noted under the subtheme of excessive concern, such as family members nagging individuals for not eating well, were viewed as obstructive by participants with T2DM. Such behaviors were associated with poorer self‐management and glycemic control.^16^ Limited knowledge and pressure to engage in certain behaviors have been demonstrated as barriers to T2DM care and can lead to increased distress and decreased adherence.^33^, ^34^ Our findings expand on existing literature in T2DM by providing insight into how social support can be helpful or harmful for individuals with GDM.^10^

### Implications for Practice and Education

There are several implications for patient education and counseling from this work. Participants viewed social support as a key aspect to managing GDM, and thus clinicians may consider integrating social support strategies with a motivational interviewing approach to promote and sustain adherence for GDM care. For example, clinicians could prompt patients to integrate children's play activities (when applicable) into their own fitness goals or inquire about family members who could join a health‐promoting activity. Clinicians may also emphasize motivators such as potential impact on the fetus. For individuals without social support, a population with poorer diabetes‐related outcomes,^35^, ^36^, ^37^ clinicians can encourage patients to consider external communal support (eg, support groups) that fit into their existing usage of online platforms. Suggestions for the incorporation of social support into GDM care are offered in Table 4. Future work can analyze the efficacy of such recommendations and tailor recommendations based on best practices in the broader T2DM literature.

**Table 4:** Implications for Clinical Practice

Theme	Suggestions
Communal support	Consider how you can help your patient integrate their own exercise routine with others’ routines or help family members pursue health goals alongside them. For example, you can ask: If there are children in your life, what are their favorite play activities? What could you do with family members to help you reach your activity goals? Do any of your family members have high blood pressure, high cholesterol, or diabetes? Consider whether support groups could be helpful, especially for patients who do not have any friends or family with diabetes. For example, you can ask: Do you use social media? What are your favorite platforms? Would you be open to an online support group? How do you use social media to support your health?
Indirect Support	Consider assessing a patient's resources for coming to and from appointments, childcare, and work accommodations. Provide referrals to community resources or social work when barriers are identified. Offer a doctor's note as well as educational tips on how to best navigate work responsibilities with GDM.
Direct Support	Consider how social support could help promote accountability toward GDM goals. For example, you can ask: Who are your biggest support systems? Do they have health and fitness goals? Do you think you could keep each other accountable? What might that look like? Consider using others’ success with their own goals as a motivator. For example, you can ask: Is there anyone is your family who was working toward a health goal and was able to achieve it? How did that make you feel? Consider counseling related to your patient's goals and hopes for this pregnancy. For example, you can ask: What are you excited about with this pregnancy? What are you hopeful for? What are your concerns about this pregnancy?
Barriers of Social Support	Consider the role social support currently plays in a patient's management of GDM. Are there any signs of stigma or limited knowledge that you can help alleviate by providing emotional support, counseling, or education resources for friends and family? For example, you can ask: How do you feel about your diagnosis? Who have you told? How did you feel when you told them? How did they react? Consider evaluating the level of social support a patient has already and to what extent that is helpful, or in some cases, overwhelming. For example, you can ask: Who are your biggest support systems right now? It sounds like they are doing an amazing job with [x]. Are there any ways they could be better at supporting you, whether that be offering more [y], or doing less of [z]?

Abbreviation: GDM, gestational diabetes mellitus.

Social support is a key social determinant of health that has been linked to a difference in health outcomes.^10^, ^12^, ^14^, ^15^, ^16^, ^31^, ^38^ This association, coupled with our findings, suggests that GDM‐focused patient education materials and interventions may be improved by leveraging the benefits of social support while preventing obstructive behaviors or misconceptions. Given the racial and socioeconomic diversity of our study population, our findings can help inform strategies to strengthen social support networks for marginalized communities, who often face unique challenges in accessing perinatal care and support.^39^, ^40^ By identifying the specific ways social support influences GDM management, our study offers insights and recommendations that can help reduce health disparities and adverse health outcomes for these patients.^39^, ^41^ Future research can focus on developing resources for patients’ families, using evidence‐based T2DM interventions as a starting point from which resources can be modified for GDM, including with a focus on people who experience the greatest health disparities. Peer support programs, cited in the T2DM literature as potentially beneficial, could be tailored to GDM.^42^, ^43^ Our findings also underscore the importance of other policy‐ and community‐based initiatives that may address the needs highlighted herein (eg, workplace accommodations).

### Strengths and Limitations

This study's strengths lie in its diverse population, including those who were Spanish‐speaking participants, had lower socioeconomic status, and had family or friends with a history of diabetes. The study's qualitative approach provides more depth and insight on social support circumstances in pregnancy and can be used as an adjunct to provide context and nuance to quantitatively focused studies in this domain. This study also had a larger sample size than many qualitative investigations. However, there are also limitations to note. This work only reflects individuals receiving care at one academic center in the context of a RCT. However, qualitative data are intended to be idea‐generating and thus are not intended to be widely generalizable.^44^

## CONCLUSION

Mobilizing a pregnant patient's social support network among those with GDM may be an important step to activate health‐promoting behaviors and promote continued medical care adherence. Such adherence is key to optimizing maternal and fetal outcomes not only in the antenatal setting, but also postpartum. Future work should further investigate how to best harness this social determinant of health while alleviating knowledge gaps or misperceptions and determine interventions that best help sustain self‐management and lifestyle change practices.

## CONFLICTS OF INTEREST

The authors have no conflicts of interest to disclose.
